# NELF Potentiates Gene Transcription in the Drosophila Embryo

**DOI:** 10.1371/journal.pone.0011498

**Published:** 2010-07-09

**Authors:** Xiaoling Wang, Saiyu Hang, Lisa Prazak, J. Peter Gergen

**Affiliations:** 1 Department of Biochemistry and Cell Biology and the Center for Developmental Genetics, Graduate Program in Biochemistry and Structural Biology, Stony Brook University, Stony Brook, New York, United States of America; 2 Graduate Program in Molecular and Cellular Biology, Stony Brook University, Stony Brook, New York, United States of America; National Institute on Aging (NIA), National Institutes of Health (NIH), United States of America

## Abstract

A hallmark of genes that are subject to developmental regulation of transcriptional elongation is association of the negative elongation factor NELF with the paused RNA polymerase complex. Here we use a combination of biochemical and genetic experiments to investigate the *in vivo* function of NELF in the Drosophila embryo. NELF associates with different gene promoter regions in correlation with the association of RNA polymerase II (Pol II) and the initial activation of gene expression during the early stages of embryogenesis. Genetic experiments reveal that maternally provided NELF is required for the activation, rather than the repression of reporter genes that emulate the expression of key developmental control genes. Furthermore, the relative requirement for NELF is dictated by attributes of the flanking cis-regulatory information. We propose that NELF-associated paused Pol II complexes provide a platform for high fidelity integration of the combinatorial spatial and temporal information that is central to the regulation of gene expression during animal development.

## Introduction

Recent findings have led to the surprising conclusion that the regulation of gene expression during animal development frequently occurs at a step downstream of the recruitment of Pol II and the initiation of transcription and involves the control of transcription elongation [1], [2], [3], [4]. A hallmark of paused Pol II complexes is their association with NELF 30–50 basepairs downstream of the transcription start site. NELF is comprised of four sub-units, NELF-A, NELF-B, NELF-D and NELF-E that are conserved from Drosophila to humans [5], [6]. In humans, low expression levels of NELF-B (also known as COBRA1, Co-factor of BRCA1) are associated with metastatic breast cancer [7]. Conversely, high expression of NELF-B and NELF-E is associated with tumorigenesis in the upper gastrointestinal tract [8], [9]. Further studies on the *in vivo* functions of NELF should help reveal the underlying molecular basis of these different diseases and provide insights on the role of regulating transcription elongation in different developmental systems.

NELF inhibits transcription *in vitro* [6], [10] and the coupling of NELF dissociation with induction of the Drosophila *hsp70* gene [5], [11] suggests NELF antagonizes transcription *in vivo*. This view is consistent with recent results indicating that Hox gene expression is antagonized by NELF in Drosophila [12]. Several studies in mammalian cells also indicate that that NELF acts to repress gene expression [13], [14], [15] and that NELF dissociation correlates with the induction of gene transcription [16]. However, other recent findings reveal the issue is more complex. Genome-wide ChIP assays reveal NELF association with the promoter regions of a large number of genes in Drosophila S2 cells, including many highly expressed genes [17]. Indeed, RNAi-mediated knockdown of NELF reduces expression of a number of genes in these cells, with a concomitant loss of chromatin architecture that is proposed to facilitate transcription [18]. This positive function is not unique to Drosophila as perturbations that increase basal transcription of *hsp70-4* in the zebrafish embryo result in increased association of NELF-A [19] and knockdown of COBRA1 in mouse embryonic stem cells leads to down-regulation of several genes [20]. These several observations clearly indicate a prominent role for NELF in the regulation of gene expression in animal systems. However, the underlying basis for NELF's participation in regulating transcription *in vivo* is clearly not understood.

In this work we use a combination of biochemical and genetic approaches to investigate the *in vivo* role of NELF in the early Drosophila embryo. We show that association of maternally provided NELF with different gene promoter regions correlates with the association of Pol II and active gene expression during the early stages of embryogenesis. Rather than seeing a loss of repression and increased expression levels in NELF-deficient embryos, our genetic experiments reveal that NELF plays a role in promoting gene expression in response to transcriptional regulators that are responsible for patterning the blastoderm embryo. Interestingly, the relative requirement for NELF depends on attributes of the flanking cis-regulatory information. Based on these results we propose that the regulatory cues that are responsible for the exquisite spatial and temporal regulation of gene expression in the Drosophila embryo are specifically integrated at the step in the transcription cycle where RNA polymerase is converted into a productive elongation complex.

## Results

### Developmental dynamics of NELF association in the Drosophila embryo

**Figure 1:**
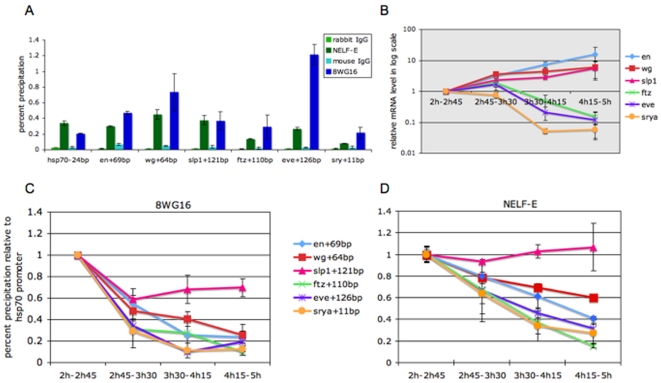
Pol II and NELF association in the early Drosophila embryo. (A) Q-PCR results on ChIP samples using antiserum against NELF-E (green bars) or the 8WG16 monoclonal antibody that recognizes Pol II (blue bars) with chromatin from 2:00–2:45 hour AED wild-type embryos. Non-specific background was determined using a control rabbit IgG (light green) or mouse IgG (light blue) antibody. Results obtained with primers for promoter-proximal regions of *hsp70*, *engrailed* (*en*), *wingless* (*wg*), *sloppy-paired-1* (*slp1*), *fushi-tarazu* (*ftz*), *even-skipped* (*eve*) and *serendipity-α* (*srya*) are as labeled from left to right across the bottom. The coordinates indicate the midpoint of the PCR product (from 150 to 205 basepairs in size) relative to the transcription start site of each gene. Error bars indicate the standard error in percent precipitation values for each interval. (B) Q-RT-PCR results on mRNA expression levels plotted on a log scale for the same five segmentation genes and *srya* with embryos from different time intervals as labeled on right. Each signal is normalized to the signal of *rp49*. (C, D) Results of Q-PCR on Pol II and NELF-E ChIP, respectively for promoter regions of the same five segmentation genes and *srya* with embryos from different time intervals as labeled in the middle. To compare the level of Pol II or NELF-association among different time windows, we assumed association of both Pol II and NELF with the *hsp70a* promoter region does not change during early embryonic development. The ChIP signal for each promoter region in a given developmental window was divided by the signal at the *hsp70a* promoter for the corresponding time window and then normalized by adjusting the ratio for the 2:00–2:45 hour AED collection to 1.0 to generate the values plotted on the Y-axis.

*In situ* hybridization and Quantitative reverse transcribed PCR (Q-RT-PCR) experiments revealed that transcripts for all four NELF subunits are provided maternally and uniformly expressed during the first 12 hours of Drosophila embryogenesis (data not shown). Chromatin Immuno-Precipitation (ChIP) experiments were done with carefully staged embryo collections from intervals spanning a time period from 2 to 5 hours after egg deposition (AED) to investigate the association of NELF with different promoter regions during early development. Quantitative PCR (Q-PCR) revealed association of NELF-E with the promoters of *hsp70a*, several segmentation genes and the cellularization gene *srya* in chromatin from embryos from the first time point, spanning from 2 hours to 2 hours and 45 minutes AED (Figure 1A). The specificity of NELF association with the promoter regions of *hsp70*, *en*, *wg* and *slp1* has been demonstrated previously [1]. Similar experiments revealed NELF is also associated specifically with the promoter regions and not with upstream regions or with the downstream transcription units of *eve*, *ftz* and *srya* (Figure S1). The association of NELF with the promoter regions of these genes strongly suggests that their early embryonic expression involves the regulation of transcription elongation.

The developmental window represented by this first time point encompasses the completion of the 13^th^ nuclear division cycle and the first half of the cellular blastoderm stage, a period during which *srya* and the pair-rule segmentation genes *eve* and *ftz* are actively expressed and during which the initial metameric expression of *en*, *wg* and *slp1* is established in response to regulation by the pair-rule transcription factors. Consistent with this, ChIP experiments revealed association of Pol II with the promoter regions of these genes at this stage of development (Figure 1A). Although the *hsp70* gene is not normally expressed at this stage, the association of both NELF and Pol II with the *hsp70* promoter is consistent with the finding that this promoter is rapidly activated in all somatic cells of blastoderm stage embryos in response to heat shock treatment [21]. Indeed, the fact that expression of *hsp70* is not developmentally regulated and that this gene remains poised for activation during subsequent developmental stages allows the association of Pol II and NELF-E with the *hsp70* promoter region to serve as a basis for normalizing results obtained with chromatin preparations from embryos collected at different developmental stages.

We performed ChIP experiments with chromatin isolated from embryos collected at three subsequent developmental time points in order to investigate the relationship between gene expression and the association of NELF and Pol II with these different promoter regions. Quantitative RT-PCR on embryos from these embryo collections revealed that expression of *srya* remained constant during the second developmental time point, corresponding to the completion of cellularization and the onset of germband extension and then fell more than 10-fold during the ensuing stages of germband extension (Figure 1B). This decline in the level of the *srya* mRNA is presaged by reduced association of Pol II and NELF-E with the *srya* promoter in embryos from the second developmental time point (Figure 1C, D). Similarly, the decline in the level of both the *eve* and *ftz* mRNAs during these later time points (Figure 1B) is also preceded by reduced association of both Pol II and NELF-E with the *eve* and *ftz* promoters (Figure 1C, D). Expression of *en*, *wg* and *slp1* mRNAs increases nearly ten-fold during these early stages (Figure 1B) as all three genes continue to be expressed in a metameric series of stripes during germband extension. Interestingly, this increase in mRNA accumulation was also found to correlate with reduced levels of Pol II and NELF-E association at these three promoters with the exception of *slp1*, which shows approximately constant levels of NELF-E association as well as the smallest reduction in Pol II association at these later developmental timepoints (Figure 1C, D). The general correlation between the association of NELF-E and Pol II with different promoters with a decline from peak levels during the blastoderm stage that occurs irrespective of whether the gene continues to be actively expressed (e.g. *en*, *wg* and *slp1*) or not (*eve*, *ftz* and *srya*) strongly suggests that the regulation of elongation is especially important during the initial phases of establishing the on/off expression patterns of these genes in the early Drosophila embryo.

### NELF has vital roles at multiple developmental stages

**Figure 2:**
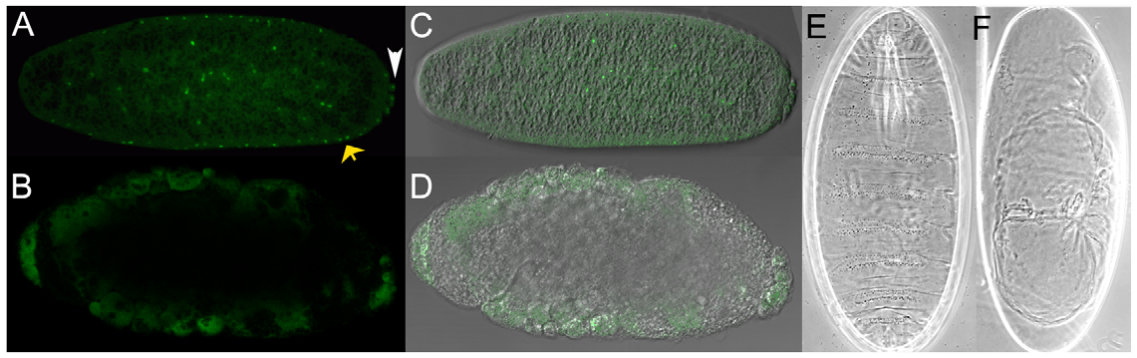
Multiple lethal phenotypes of NELF-deficient embryos. Nuclear morphology of representative embryos from 2–4 hour AED collections of wild-type (A) and *NELF-A[KG]* GLC females (B) as revealed by Pico Green staining. Nuclei in the wild-type embryo were apparent in the posterior pole cells (white arrowhead) and apical cytoplasm (yellow arrows), with some remaining in the central yolk. The cytoplasmic disorganization of NELF-A deficient embryos was evident in the phase contrast overlay images of these embryos (C, D). Most *NELF-A* GLC embryos displayed this early arrest phenotype (111 of 171 embryos from a fixed 0–14 hour AED collection). NELF-A deficient embryos that make it to the blastoderm stage gastrulated and underwent normal germband extension but then arrested during germband retraction. The defects in head development and incomplete dorsal closure caused by loss of maternal NELF-A were apparent in cuticle preparations of unhatched wild-type (E) and *NELF-A* GLC (F) embryos. *NELF-A* GLC embryos rarely (<1%) hatched, with the surviving larvae developing to fertile adults. The same range of phenotypes was obtained in *NELF-E* GLC embryos, with a lower frequency of early arrest (111 of 481 embryos in a >24 hour AED collection) and a larval hatch rate of over 50% (288 of 481 embryos allowed to develop for 24 hours).

We used transposon insertion mutations in the *NELF-A* and *NELF-E* genes to investigate the *in vivo* function of NELF. Flies heterozygous for the *NELF-A[KG]* transposon insertion and a deficiency chromosome that removes the *NELF-A* locus appear morphologically normal at the end of embryogenesis and hatch as 1^st^ instar larvae. Although these larvae survive for several days they do not increase in size, indicating an essential role for *NELF-A* in post-embryonic development. The observation that *NELF-A* mutant embryos survive without gross patterning defects is not surprising based on the findings presented above that NELF-A is maternally provided. To investigate the role of NELF during embryogenesis we generated female germ cells homozygous for the *NELF-A[KG]* mutation. Q-RT-PCR fails to detect *NELF-A* transcripts in 0–1 hour AED *NELF-A[KG]* germline clone (GLC) embryos. A low level, less than 1% of that in wild-type is detected in blastoderm stage embryos presumably due to zygotic expression of a paternally inherited wild-type *NELF-A* allele. More than half of NELF-A deficient embryos arrest prior to the cellular blastoderm stage and display abnormal nuclear morphology (Figure 2A–D). This phenotype is reminiscent of the enlarged and multinucleate phenotype of HeLa cells depleted for NELF-E [22]. Embryos that escape this early arrest proceed through the blastoderm stage and gastrulate normally, but then almost always arrest during germband retraction with head defects and incomplete dorsal closure (Figure 2E, F).

**Figure 3:**
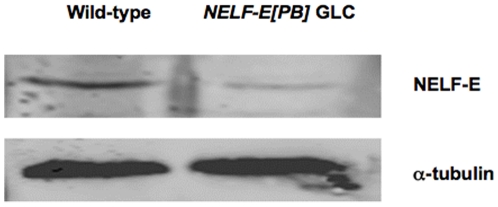
NELF-E Expression in NELF-E[PB] germline clone embryos. Western blots were done on 0–1 hour AED collections of wild-type and *NELF-E[PB]* GLC embryos to determine whether the transcripts expressed in the GLC embryos produced protein. The Western blot shown in this figure indicates reduced levels of NELF-E relative to α-tubulin in the *NELF-E[PB]* GLC embryos.

Similar experiments were carried out with the *NELF-E[PB]* mutation to investigate the developmental requirements for maternally provided NELF-E. In this case Q-RT-PCR indicates an approximate 5-fold reduction of *NELF-E* transcript levels in pre-blastoderm *NELF-E[PB]* GLC embryos. Consistent with this reduced expression of maternal transcripts, NELF-E protein was detected at reduced levels in these embryos (Figure 3). Both the early and late arrest phenotypes are observed in *NELF-E[PB]* GLC embryos, with a decrease in the proportion of embryos that arrest prior to the blastoderm stage to about 25%, and an increase in the proportion of viable larvae that hatch to more than 50%. The finding that both phenotypes of arrested embryos are obtained in embryos that lack maternally provided NELF-A as well as in embryos with reduced levels of maternal NELF-E is strong evidence that these phenotypes result from the reduced activity of the NELF complex.

### A facultative role for NELF in promoting gene expression

**Figure 4:**
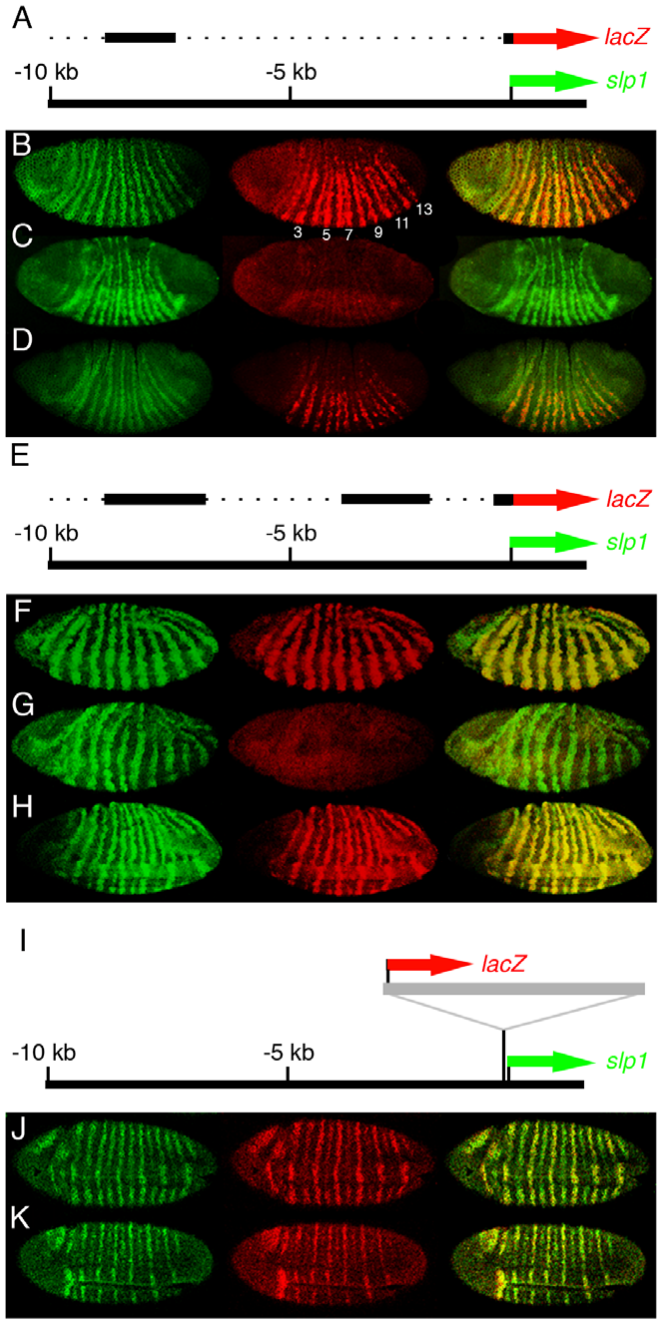
Differential requirements for NELF in expression of slp1-lacZ reporter genes. Fluorescent double *in situ* hybridization was used to compare the expression of the endogenous *slp1* (green) and *lacZ* (red) mRNAs in embryos of different genotypes. Embryos are oriented anterior to the left, dorsal side up. (A) Schematic diagram of the *slp1[DESE]-lacZ* reporter gene, containing a DNA segment that extends from 8.7 to 7.2 kb upstream of *slp1* fused to a 129 bp *slp1* basal promoter segment followed by the *E. coli lacZ* structural gene. The solid black line represents the *slp1* locus with coordinates given at positions 5 and 10 kb upstream of the promoter. DNA segments included in the reporter transgene are shown as solid lines above this map, with the dotted line indicating flanking DNA that is omitted from the transgene. (B) Expression of endogenous *slp1* (green) and the *slp1[DESE]-lacZ* reporter (red) in a gastrula stage wild-type embryo. The merged image (rightmost column) demonstrates that expression from the reporter gene overlaps with *slp1*, although the odd-numbered stripes (numbered in white) were somewhat stronger, with ectopic *lacZ* expression anterior to the odd stripes (most apparent anterior to stripes 5 and 7). (C) *NELF-A[KG]* GLC embryos had relatively normal *slp1* expression, but lost *slp1[DESE]-lacZ* expression. (D) Expression from this reporter was reduced but not eliminated in *NELF-E[PB]* GLC embryos. (E) *slp1[DESE+PESE]-lacZ* contains DNA segments from 8.7 to 6.6 and from 3.9 to 1.8 kb upstream of the *slp1* promoter fused to a 381 bp segment spanning the *slp1* promoter and then *lacZ*. (F) This reporter faithfully recapitulated *slp1* expression throughout the segmented region of a gastrula stage embryo with the only obvious difference being the absence of a stripe of expression in the anterior head region. (G) Expression of *slp1[DESE+PESE]-lacZ* was not detected in *NELF-A[KG]* GLC embryos that had relatively normal *slp1* expression. (H) This same reporter was expressed and recapitulates *slp1* expression in a gastrula stage *NELF-E[PB]* GLC embryo. (I) *P{PZ}slp1[05965]* is an enhancer trap insertion inserted 44 base-pairs upstream of *slp1* transcription start site. Expression of *lacZ* mRNA from this transposon faithfully recapitulated *slp1* expression at the gastrula stage in both wild-type (J) and *NELF-A[KG]* GLC (K) embryos.

We used *in situ* hybridization to examine gene expression in NELF-deficient embryos. Somewhat surprisingly we found no overt changes in the blastoderm stage expression of several different segmentation genes in embryos that lack maternal NELF-A (see below). In order to further probe the potential involvement of NELF in transcriptional regulation at this stage we took advantage of a reporter gene containing an upstream cis-regulatory element from the NELF-associated *slp1* gene (Figure 4A). The *slp1[DESE]-lacZ* reporter drives early expression of both the odd- and even-numbered stripes, but also fails to be fully repressed in the anterior regions of the odd-numbered parasegments (Figure 4B). We reasoned that this reporter might be especially sensitive to a loss of NELF-dependent repression. Contrary to this expectation, we found expression of the *slp1[DESE]-lacZ* reporter is nearly eliminated in *NELF-A* deficient embryos that show relatively normal expression of endogenous *slp1* mRNA (Figure 4C). Consistent with this result, we found a reduction, but not total elimination of *slp1[DESE]lacZ* expression in embryos with reduced levels of maternally provided NELF-E (Figure 4D). A partial loss of expression in *NELF-E* GLC embryos was similarly observed with *slp1[PESE]-lacZ*, a second reporter gene containing a distinct upstream segment of *slp1* cis-regulatory DNA that drives expression only in even-numbered parasegments (Figure S2). From these results we concluded that NELF contributes to the expression of these reporter genes in a manner that is sensitive to NELF dosage. Importantly, the observation that endogenous *slp1* expression was relatively normal in these same embryos indicates the defects in reporter gene activation were not an indirect consequence of gross perturbations in embryonic metabolism.

The *slp1[DESE]-lacZ* and *slp1[PESE]-lacZ* reporters each generate an incomplete expression pattern. In order to further investigate the differential requirements for NELF in the expression of these reporter genes versus the endogenous *slp1* locus we examined the expression of a reporter gene that more faithfully recapitulates endogenous *slp1* expression. The *slp1[DESE+PESE]-lacZ* transgene, containing a larger basal promoter and both segments of *slp1* upstream cis-regulatory DNA (Figure 4E), drives 14 stripes of *lacZ* expression with restored inter-stripe repression in odd-numbered parasegments of wild-type embryos (Figure 4F). Expression of *slp1[DESE+PESE]-lacZ* was also greatly reduced in *NELF-A[KG] GLC* embryos (Figure 4G), but was not as significantly affected in embryos with reduced NELF-E levels (Figure 4H). The reduced sensitivity of the composite *slp1[DESE+PESE]-lacZ* reporter to NELF-E depletion suggests that NELF makes a quantitative contribution to transcription that can be superseded by flanking cis-regulatory information. As a further test of this idea we examined expression of *P{PZ}slp1[05965]*, an enhancer trap P-transposon inserted 44 basepairs upstream of the *slp1* promoter (Figure 4I). Transcription of *lacZ* mRNA from this enhancer trap transposon initiates at the P-element promoter in response to endogenous *slp1* cis-regulatory DNA and faithfully recapitulates the full *slp1* expression pattern in gastrula stage embryos (Figure 4J). In contrast to reporter genes containing only defined subsets of flanking cis-regulatory DNA from the *slp1* locus, the enhancer trap was expressed in NELF-A deficient embryos (Figure 4K). This result not only provided additional evidence that the requirement for NELF is dependent on attributes of the flanking cis-regulatory DNA, but also rules out explanations based on differences in the processing or stability of the *slp1* and *lacZ* mRNAs.

**Figure 5:**
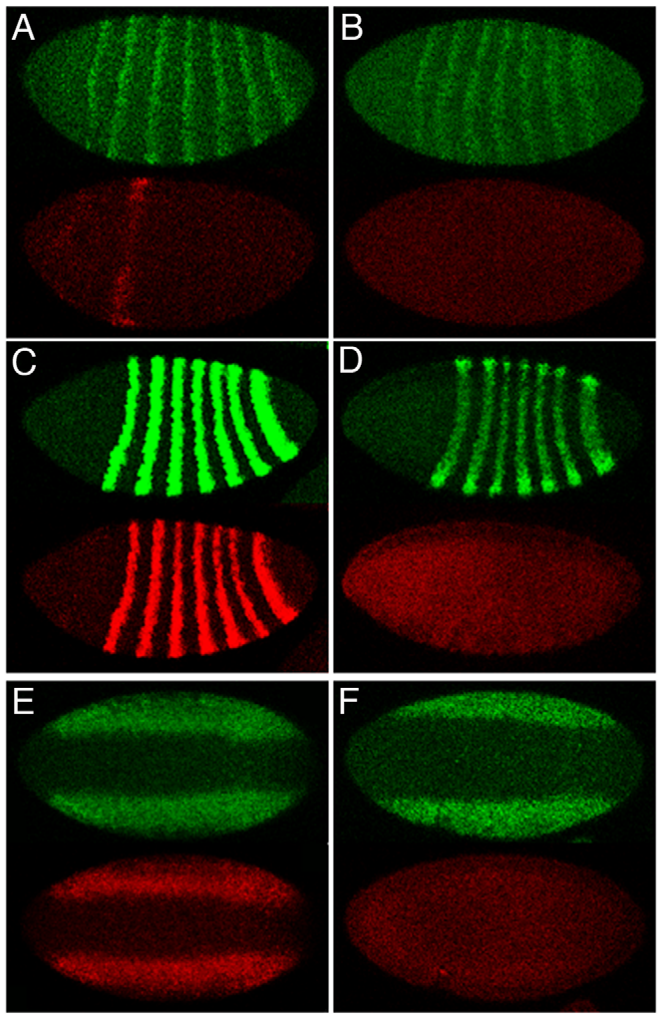
NELF-dependent activity of cis-regulatory elements that mediate blastoderm patterning. Whole-mount *in situ* hybridization revealed the expression of *eve*, *ftz* and *rho* mRNAs relative to the expression of *lacZ* reporter genes that emulated aspects of the blastoderm stage expression of the endogenous genes. In each case, expression of the endogenous gene is shown in green, and *lacZ* expression is shown in red. Expression of *eve* and *ftz* is best visualized in embryos oriented as in Figure 3, whereas an *en face* ventral view most clearly reveals expression of dorsal-ventral patterning genes such as *rho*. *P{MSE-lacZ}* was expressed in cells corresponding to stripe #2 of the pair-rule gene *eve* in wild-type embryos (A), but failed to be expressed in *NELF-A[KG]* GLC embryos that showed pair-rule expression of the endogenous gene (B). *P{ftz/lacC}* was expressed in a pair-rule pattern similar to *ftz* in wild-type embryos (C), but failed to be expressed in *ftz*-expressing NELF-A deficient embryos (D). The *P{Dm rho[NEE]-lacZ}* transgene faithfully emulated the early activation of *rho* in the neurogenic ectoderm in wild-type embryos (E), but was not expressed in embryos that lacked maternal NELF-A (F). The intensity of expression of the endogenous loci was somewhat variable in NELF-A deficient embryos, with occasional defects in patterning. Additional experiments not presented here revealed a similar lack of overt changes in the expression of the segmentation genes *runt*, *hairy*, *odd*, *en*, and *wg* in embryos that lacked maternal NELF-A.

In order to determine if NELF-dependence is restricted to the *slp1* reporters we examined the expression of reporters that emulate aspects of the blastoderm stage expression of other genes involved in embryonic pattern formation. NELF-A deficient embryos failed to express reporters containing the minimal element for stripe #2 of the *even-skipped* gene, the 6.3 kb upstream element in the *ftz-LacC* reporter, and the NEE element of the dorsal-ventral patterning gene *rhomboid* (Figure 5). As was found for *slp1*, the expression of each of the endogenous cognate genes was relatively normal in these same embryos. These results indicate that the requirement for NELF is revealing a common functional distinction between the properties of these several different reporter genes and the endogenous chromosomal loci.

## Discussion

### Cis-regulatory DNA and NELF-dependent transcription

A principle conclusion that emerges from these results is that NELF can play a positive role in supporting transcription in the Drosophila embryo. This finding is somewhat surprising based on NELF's well-characterized properties as a transcriptional inhibitor *in vitro* and the current view of its role in regulating the *hsp70* gene *in vivo*. So how does a factor that antagonizes transcription elongation play a positive role in promoting gene expression? Depletion of NELF in Drosophila S2 cells leads to reduced expression of a number of genes, and this drop in expression levels correlates with the re-positioning of nucleosomes around the promoter [18]. The idea that NELF stabilizes the local architecture at the promoter that supports transcription is attractive, but our results indicate these presumptive architectural contributions are not essential for transcription of several endogenous loci in the early embryo.

Central to understanding the requirement for NELF in promoting transcription is defining the key differences between the endogenous *eve*, *ftz*, *rho* and *slp1* loci and the NELF-dependent reporter genes containing different specific cis-regulatory enhancers from these genes. Our results strongly suggest that it is not the basal promoter *per se* that dictates the requirement for NELF. The basal promoter region contained in the composite *slp1[DESE+PESE]-lacZ* reporter extends from 260 bp upstream to 121 bp downstream of the transcription start site and includes the entire 5′ untranslated region of the *slp1* mRNA. Even more telling is the observation that expression of the *ftz-lacC* reporter is lost in *NELF-A* GLC embryos. This reporter contains 6.5 kb of contiguous upstream cis-regulatory DNA extending to 120 bp downstream of the transcription start site. This stands in contrast to the NELF-independent expression of the *P{PZ}slp1[05965]* enhancer trap inserted 44 bp upstream of the *slp1* transcription start site. Transcription of *lacZ* mRNA from this enhancer trap presumably initiates at the P-element promoter located at the 5′ terminus of this transgene insertion.

Although the requirement for NELF appears not to be dictated by the basal promoter, the observations that NELF is specifically localized to promoter regions and does not travel with elongating Pol II complexes [1], [5], [14], [17] strongly suggest the requirement involves NELF-associated Pol II complexes paused downstream of the promoter. The differential effect of NELF-E depletion on expression of the different *slp1-lacZ* reporters further indicates that the relative requirement for NELF is a function of the extent of flanking cis-regulatory information. Taken together these observations suggest that the relative requirement for NELF depends on interactions involving these flanking cis-regulatory DNA regions and NELF-associated paused Pol II complexes. We propose that NELF interacts with Pol II complexes that have initiated transcription but that are not fully competent to enter productive elongation and helps to stabilize these complexes in a form that is competent for responding to activating (or repressing) cues from enhancer-bound transcription factors. In this model the relative requirement for NELF in allowing for active transcription would depend on the strength of the interaction between a promoter and an enhancer and the relative efficiency of generating productive elongation complexes. Although the enhancers contained in the different *lacZ* reporters used in our experiments are all clearly capable of communicating with the promoter it would certainly be expected that this communication would be less efficient than for endogenous loci that contain the full complement of flanking DNA that has evolved to optimize the regulation of gene expression at this stage. Importantly, the NELF-dependent expression of these reporters strongly suggests that the generation of a productive Pol II elongation complex is the key step in the transcription cycle that is targeted for integrating the regulatory cues that drive the patterned expression of these genes in the early embryo.

### Developmental role of NELF

NELF clearly has a pleiotropic role during Drosophila development. Perturbations in maternally provided NELF lead to two distinct embryonic lethal phenotypes. The observation that both phenotypes, albeit with different penetrance are produced either by elimination of maternal NELF-A or by reduction of maternal NELF-E strongly suggests both phenotypes are due to decreased activity of the NELF complex. The early arrest phenotype occurs prior to the onset of transcription in the embryo and thus is most likely due to defects that occur during oogenesis. The maternally provided histone mRNAs are one likely candidate as a prospective target of NELF activity during oogenesis. NELF is required for the proper processing of the 3′ termini of replication-dependent histone mRNAs in HeLa cells [23], and Drosophila embryos with defects in the processing of maternally provided histone mRNAs arrest during the nuclear division cycles that precede the blastoderm stage [24]. Further studies should reveal whether the early arrest of NELF-A and NELF-E GLC embryos reflects a conserved role for NELF in the 3′-end processing of histone mRNAs.

The finding that many genes have paused Pol II complexes at their 5′ end [2], [17] strongly suggests that the regulation of transcription elongation is a widespread phenomenon in higher eukaryotes. Recent studies indicate that more than one third of all genes in Drosophila S2 cells generate short, 5′-capped RNAs similar to those produced by stalling of Pol II [25]. The results of Pol II chromatin immunoprecipitation whole genome microarray assays suggest that paused Pol II complexes are formed on approximately 10% of genes in the blastoderm stage Drosophila embryo [4]. This is almost certainly an under-estimate as five of the seven genes for which we have demonstrated NELF association were not identified as having paused Pol II complexes at this stage. Indeed, the stringent cut-off used in this study led to the assignment of *slp1* as a member of the 27% of genes that have uniform Pol II association in the blastoderm embryo.

It is furthermore clear that NELF association is developmentally regulated as neither *srya* nor any of the five segmentation genes for which we demonstrate NELF association in the early embryo are also associated with NELF in S2 cells [17]. Amongst these six genes with early embryonic association of NELF there are differences in the level of association at different developmental stages. The two genes with the most rapid loss of NELF, *ftz* and *srya* show little to no expression after four hours of development [26], [27]. Thus NELF is not involved in the stable maintenance of repression at these later stages, which involves instead other mechanisms such as epigenetic maintenance by the Polycomb group proteins and specific histone methylation marks [28], [29], [30]. The observation that NELF association is also reduced on genes such as *en* and *wg* that have increased expression levels at later stages may suggest that NELF is not involved in the ongoing expression of these genes at later stages. However, as the embryo is comprised of a mixture of expressing and non-expressing cells it will be important to examine NELF association specifically in cells expressing these genes before coming to this conclusion.

The high levels of NELF association with the promoter regions of a number of genes involved in segmentation and other early developmental processes serves to emphasize the unique and pivotal aspects of this critical stage of Drosophila embryogenesis. Pre-blastoderm nuclei are totipotent and come to be specified in response to maternally-provided positional information and the action of the genetic systems that respond to this information. The regulation of gene transcription is central to the initial specification of cell fates along both the anterior-posterior and dorsal-ventral axes of the early embryo, and it is clear that regulation of transcription elongation is central to this process. Similar to Drosophila blastoderm nuclei, the pluripotent properties of human embryonic stem cells are reflected by the presence of paused Pol II complexes on a wide number of genes, including many key developmental regulators [2]. Further studies on the mechanisms of developmentally regulated transcription elongation are clearly of great importance for understanding the initial programming of cell fates expression during animal embryogenesis.

## Materials and Methods

### Western blot and ChIP

Rabbit Anti-NELF-A and anti-NELF-E antibodies were provided by David Gilmour [11]. Normal rabbit IgG and anti-rabbit IgG were from Sigma. Normal mouse IgG and the monoclonal antibody 8WG16 that recognizes RNA polymerase II were obtained from Santa Cruz and Covance, respectively. Embryos for ChIP were collected for 45 minutes and aged for 2:00, 2:45, 3:30 and 4:15 hours prior to fixation, respectively. Prior to fixation, overaged embryos were removed from the embryo collections after examination under a microscope. ChIP was performed as described previously [1]. Each ChIP experiment was repeated twice using independent chromatin preparations. Primer sequences are available on request.

### Drosophila strains and transgenes

The *P{SUPorP}[KG09483]* transposon insertion (hereafter referred to as *NELF-A[KG]*) is located in the first intron of *NELF-A* [31]. The chromosome carrying the *NELF-A[KG]* mutation obtained from the Bloomington Stock Center carries an additional lethal mutation as precise excision of the transposon fails to revert the recessive lethal phenotype of the chromosome. *NELF-A* is indeed vital as the *NELF-A[KG]* mutation is lethal over *Df(3R)e-R1*, a deficiency that removes the locus, and this lethality is reverted by transposon excision. The extraneous lethal mutation was removed by meiotic recombination to generate the *P{neoFRT}82B cu[1] sr[1] NELF-A[KG]* chromosome used in this work. In the case of *NELF-E*, the recessive lethal phenotype of the *PBac{PB}[c00768]* insertion (hereafter referred to as *NELF-E[PB]*) in the first intron of *NELF-E* [32] is suppressed in flies that carry an *hsp83-NELF-E* transgene (D. Gilmour, personal communication). A recombinant *NELF-E[PB] st[1] P{FRT(w[hs])}2A sr[1]* chromosome was generated for this work.

The *P{y+slp1[8772]-lacZ att}29* transgene, referred to as *slp1[DESE]lacZ* in the text contains sequences from 8.7 to 7.2 kilobase-pairs (kb) upstream of the *slp1* promoter followed by a segment that extends from 72 basepairs (bp) upstream to 57 bp downstream of the *slp1* transcription start site inserted into a CaSpeR-AUG-β-gal vector [33] that also contains an attB recognition site inserted in the PstI restriction site downstream of the *lacZ* gene. This transgene was integrated into the *P{CaryP}attP2* site by coinjection of the plasmid with mRNA encoding the ΦC31 integrase [34]. The *P{y+slp1[3918]-lacZ att}32* transgene ( = *slp1[PESE]-lacZ*) is similar to *slp1[DESE]-lacZ*, but with an upstream cis-regulatory DNA segment that extends from 3.9 to 1.8 kb upstream of the *slp1* promoter. The *P{w+slp1[8765/3918]-lacZ}7.2* transgene (*slp1[DESE+PESE]*-lacZ) includes *slp1* upstream DNA from 8.7 to 6.5 kb and from 3.9 to 1.8 kb followed by a basal promoter segment that spans from 260 bp upstream to 121 bp downstream of the transcription start site inserted in a derivative of CaSpeR-AUG-β-gal obtained from Miki Fujioka (Thomas Jefferson University) that contains Glass binding sites inserted upstream of mini-*white* [35]. *P{PZ}slp1[05965]*, a *rosy*-based enhancer trap transposon inserted 44 bp upstream of the *slp1* transcription start site expresses *lacZ* from the *P*-element promoter in the same 5′ to 3′ direction as the downstream *slp1* transcription unit [36].

The *P{MSE-lacZ}* transgene contains the *eve* minimal stripe 2 element, from 1.55 to 1.1 kb upstream of the promoter, fused to sequences extending from −42 to +160 bp of the *eve* promoter [37]. The *P{ry[+t7.2] = ftz/lacC}1* reporter gene transposon contains a 6.5 kb segment spanning the *ftz* upstream, neural and zebra elements and basal promoter sequences extending through the entire 120 bp 5′-untranslated region [38]. The *P{Dm rho[NEE]-lacZ}3* transgene (*rho[NEE]-lacZ*) contains an 871 bp segment of *rho* upstream DNA inserted into the [-42EvelacZ]-pCaSpeR transformation vector [39].

### Germline clone experiments

Mitotic recombination using the *FLP/FRT/ovo[D]* system [40] was used to generate female germ cells homozygous for the *NELF-A[KG]* and *NELF-E[PB]* mutations. For *NELF-A*, females homozygous for the X-linked *y w P{hsFLP}22* chromosome and heterozygous for the recombinant *P{neoFRT}82B cu[1] sr[1] NELF-A[KG09483]* chromosome and a *TM3* balancer were mated to *P{neoFRT}82B P{OvoD1-18}3R/TM3, Sb[1]* males. Progeny from this cross were heat-shocked at 37°C for 1 hour on two consecutive days starting 24 hours AED. Female progeny from this cross heterozygous for the *P{neoFRT}82B cu[1] sr[1] NELF-A[KG09483]* and *P{neoFRT}82B P{OvoD1-18}3R* chromosomes were collected and mated to males of different genotypes to generate embryos lacking maternally provided *NELF-A*. A similar protocol was used for *NELF-E* but involved heat-shocking progeny from a cross between females heterozygous for the *NELF-E[PB] st[1] P{FRT(w[hs])}2A sr[1]* chromosome and *P{ovoD1-18}3L P{FRT(w[hs])}2A/TM3, Sb[1]* males. The phenotype of embryos that arrest prior to cellularization was characterized by staining a 2–4 hour AED collection of embryos with Pico Green. Cuticle preparations on embryos that were allowed to develop for more than 24 hours were done by transferring dechorionated embryos into Lacto∶Hoyers (1∶1) on a microscope slide and incubating the slide overnight at 60°C.

### *In situ* hybridization and RT-PCR

The *in situ* hybridization protocol for detection of different mRNAs with fluorophore conjugated antibodies was essentially as described by Janssens et al [41]. Embryos were collected from crosses between *NELF-A* or *NELF-E* GLC females and reporter gene-bearing males. Homozygous males were used for the *slp1[DESE]*, *slp1[DESE+PESE]*, *P{MSE-lacZ}*, and *P{Dm rho[NEE]-lacZ}3* reporters, whereas heterozygous males were used for the *P{PZ}slp1[05965]* enhancer trap transposon and the *P{ry[+t7.2] = ftz/lacC}1* reporter gene which is carried on a *CyO* balancer. As a control for the wild-type expression of these out-crossed reporter genes, embryos were collected from a cross between *y w*; *P{CaryP}attP2* females and reporter gene-bearing males. The *lacZ* riboprobe was generated using the Fluorescein RNA labeling mix (Roche), whereas riboprobes for *slp1*, *eve*, *ftz*, *rho* and other endogenous mRNAs were generated using the Digoxigenin RNA labeling mix (Roche) [42]. The linearized DNA templates and RNA polymerases used for synthesis of the *lacZ*, *eve*, *ftz* and *slp1* riboprobes are as described previously [43], [44]. The *rho* riboprobe was made using T7 RNA Polymerase from EcoRI-digested cDNA clone LD01631 (Drosophila Genomics Resource Center). After hybridization, *lacZ* mRNA was visualized by sequential incubation with Rabbit Anti-fluorescein (1µg/ml final) and Alexa Fluor 647 Donkey Anti-rabbit (1µg/ml) antibodies (Molecular Probes). Digoxigenin labeled probes were detected using Mouse Anti-Digoxigenin antibody (Roche, 1.25 µg/ml final) followed by Alexa Fluor 555 Goat Anti-mouse (1µg/ml) and Alexa Fluor 555 Donkey Anti-Goat (1µg/ml) antibodies (Molecular Probes). Embryos were mounted in 2.5% Dabco (Sigma), 50 mM Tris (pH 8.0), 90% glycerol and were imaged on a Leica TCS SP2 Spectral Confocal Microscope system with non-overlapping wavelength windows of 560–645 and 650–715 nm, respectively.

RNA used for Q-RT-PCR was isolated from homogenates of 200 appropriately staged embryos. RNA was extracted using the High Pure RNA isolation Kit (Roche). cDNA was synthesized with the Quanta Biosciences qScript cDNA synthesis kit programmed with 1ug of RNA. Quantitative PCR was conducted with primer pairs centered 500 to 700bp downstream of each gene. RT-PCR signal obtained from different time intervals was normalized using the RT-PCR signal for rp49. Primer sequences are available on request.

## Supporting Information

Figure S1 Supplemental Figure S1
(0.04 MB DOC)

Figure S2 Supplemental Figure S2
(0.52 MB DOC)
