# Supplementary material for: NELF Potentiates Gene Transcription in the Drosophila Embryo
Source: PLoS One. 2010 Jul 9;5(7):e11498. doi: 10.1371/journal.pone.0011498 (PMC2901382; doi:10.1371/journal.pone.0011498)
Supplement: Figure S1 — Supplemental Figure S1 (0.04 MB DOC) [file pone.0011498.s001.doc]

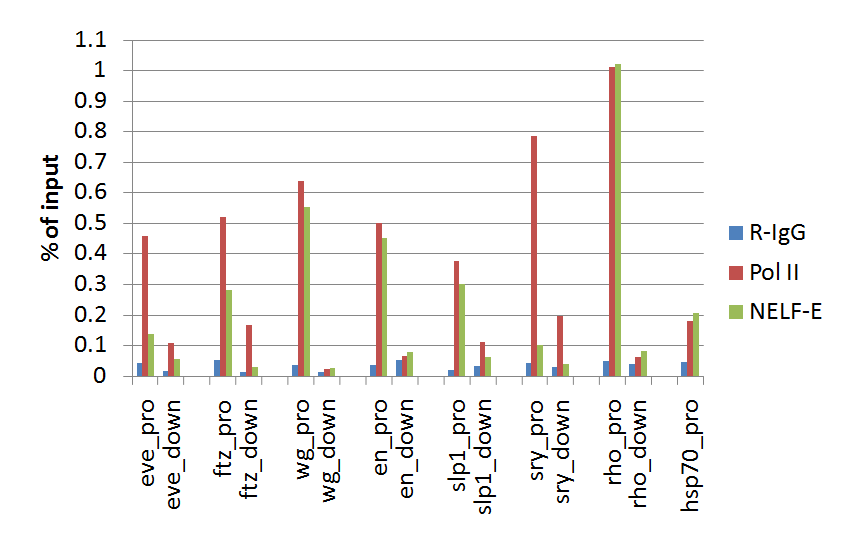


***Figure S1. NELF specifically associates with promoter proximal regions.***

This graph shows a plot of the ChIP signals obtained with antisera against RNA polymerase II (red bars) and NELF-E (green bars) as well as a background control using rabbit IgG (blue bars). Results with two different primer pairs, one pair near to promoter and a second pair centered more than 500 bp downstream of the transcription start site are shown for seven different genes as labeled across the bottom. These experiments were conducted using chromatin from 2:45 – 3:30 AED embryos. For each gene, the NELF-E signal is stronger with the promoter proximal primer pair, and the downstream signal is close to background levels. In contrast, and as expected for genes that are expressed at this developmental stage, Pol II ChIP signals above background are obtained with the downstream primer pair for *eve*, *ftz*, *slp1*, and *srya.*
