# Supplementary material for: NELF Potentiates Gene Transcription in the Drosophila Embryo
Source: PLoS One. 2010 Jul 9;5(7):e11498. doi: 10.1371/journal.pone.0011498 (PMC2901382; doi:10.1371/journal.pone.0011498)
Supplement: Figure S2 — Supplemental Figure S2 (0.52 MB DOC) [file pone.0011498.s002.doc]

**
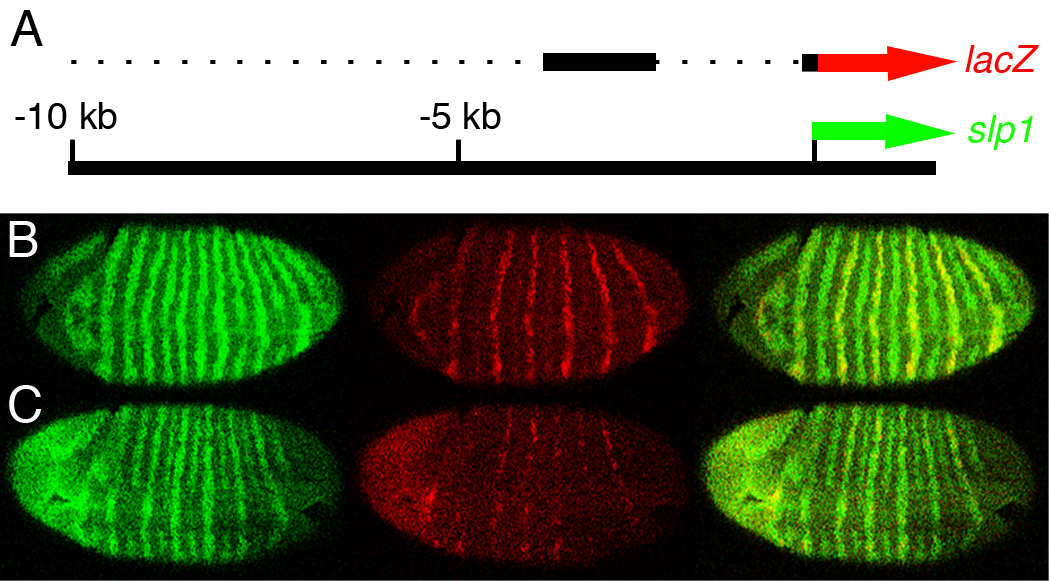
**

***Figure S2. NELF-E potentiates expression of the slp1[PESE]-lacZ reporter.*** Fluorescent double *in situ* hybridization was used to compare the expression of a reporter gene containing a *slp1* cis-regulatory element extending from 3.9 to 1.8 kb upstream of the *slp1* promoter fused to a 129 bp *slp1* basal promoter, followed by *lacZ* with that of the endogenous *slp1* mRNA (green). (A) schematic diagram of the *slp1[PESE]-lacZ* reporter, labeled as in Figure 4. (B) *lacZ* expression (red) in wild-type gastrula stage embryos is observed only in cells corresponding to the even-numbered *slp1* stripes. (C) reporter gene expression is reduced in *NELF-E[PB]* germline clone embryos.
